# Total Chemical Synthesis of Modified Histones

**DOI:** 10.3389/fchem.2018.00019

**Published:** 2018-02-06

**Authors:** Yun-Kun Qi, Hua-Song Ai, Yi-Ming Li, Baihui Yan

**Affiliations:** ^1^Department of Anesthesiology, The Second Affiliated Hospital of Xi'an Jiaotong University, Xi'an, China; ^2^Department of Medicinal Chemistry, School of Pharmacy, Qingdao University, Qingdao, China; ^3^Department of Chemistry, Tsinghua University, Beijing, China; ^4^Department of Pharmacy, School of Biological and Medical Engineering, Hefei University of Technology, Hefei, China

**Keywords:** histones, post-translational modifications, protein chemical synthesis, total chemical synthesis, native chemical ligation

## Abstract

In the post-genome era, epigenetics has received increasing attentions in recent years. The post-translational modifications (PTMs) of four core histones play central roles in epigenetic regulation of eukaryotic genome by either directly altering the biophysical properties of nucleosomes or by recruiting other effector proteins. In order to study the biological functions and structural mechanisms of these histone PTMs, an obligatory step is to prepare a sufficient amount of homogeneously modified histones. This task cannot be fully accomplished either by recombinant technology or enzymatic modification. In this context, synthetic chemists have developed novel protein synthetic tools and state-of-the-art chemical ligation strategies for the preparation of homologous modified histones. In this review, we summarize the recent advances in the preparation of modified histones, focusing on the total chemical synthesis strategies. The importance and potential of synthetic chemistry for the study of histone code will be also discussed.

## Introduction

**Figure 1:**
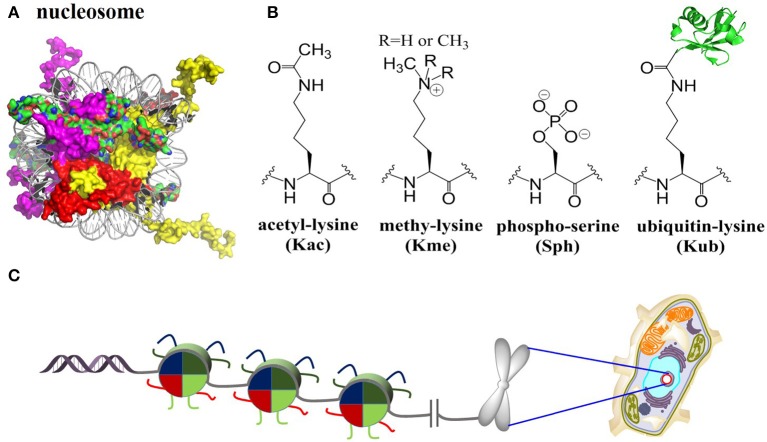
Histones nucleosomes and chromatin. **(A)** Structure of the nucleosome core particle [Protein Data Bank (PDB) number 1KX5]: Histones are color-coded as follows: H2A: red, H2B: purple, H3: yellow, H4: green. **(B)** Selected four histone PTMs: lysine acetylation, lysine methylation (mono/di/tri), serine phosphorylation, and lysine ubiquitination. **(C)** Packaging of genomic DNA into nucleosomes arrays and chromosomes in eukaryotic nucleus.

In eukaryotic cells, chromatin encodes epigenetic information and controls the stability of the genome (Luger et al., 1997). The fundamental DNA-packaging unit of chromatin is the nucleosome core particle (NCP), which consists approximately 147 base pairs of DNA wrapping twice around a histone octamer containing two copies of each core histone protein (known as H2A, H2B, H3, and H4) (Figure 1). NCP assembly is believed to be a pivotal and highly regulated procedure that restores structure and function of chromatin in DNA replication, gene transcription, DNA damage repair, etc., (Muller and Muir, 2015; Sewitz et al., 2017). The four core histone proteins are site-specifically modified by complex post-translational modifications (PTMs), including methylation (mono, di and tri), acetylation, phosphorylation, ubiquitination, SUMOylation and glycosylation (Figure 1B) (Suganuma and Workman, 2008; Huang et al., 2015). These PTMs are covalently introduced, recognized/interpreted, and removed by a cascade of enzymes and proteins named “writers,” “readers,” and “erasers” (Santos-Rosa et al., 2002; Lee et al., 2005; Kim et al., 2009; Wu et al., 2011; Mattiroli et al., 2012; Fradet-Turcotte et al., 2013; Patel and Wang, 2013; Muller and Muir, 2015; Morgan et al., 2016; Wang et al., 2016b).

Through directly altering the biophysical properties of nucleosomes or recruitment of effector proteins, these PTMs regulate gene expression and a series of DNA-related processes within chromatin (Muller and Muir, 2015). The study of complicated PTMs as well as their biological function and mechanism of action raised the “histone code” hypothesis (Strahl and Allis, 2000; Jenuwein and Allis, 2001), which has profound effects on the cognition of DNA-related processes and treatments of diseases such as cancer (Chi et al., 2010; Maze et al., 2014). However, due to the diversity of PTM sites, complexity of PTM types as well as the synergies of multiple PTMs, it remains largely unknown how these PTMs are mediating epigenetic regulation (Maity et al., 2016). To elucidate the biochemical and structural mechanisms of various PTMs as well as how these PTMs mediate epigenetic regulation, it is essential to obtain sufficient amount of homogeneously modified histones with site-specific PTMs (Maity et al., 2016).

## Current methods for generation of modified histones

Modified histones could be obtained through gene expression methods which rely on the purification of histone proteins with PTMs. However, due to the diversity of PTM sites and PTM types, it is considered as a challenging task to obtain homogeneously modified histones through traditional recombinant strategies (Maity et al., 2016). Enzymatic methods, which depend on the enzymatic modification of the unmodified histones, could also be used to obtain modified histones (Basnet et al., 2014). However, the enzymes (often protein complexes) responsible for specific modification may be either still unknown or difficult to reconstruct (Tang et al., 2013). Moreover, even if the enzymes could be obtained in their active forms, the enzymes may modify the native histones heterogeneously *in vitro*. At present, one successful example of enzymatic modified histones is a mono-ubiquitination of H2A at Lys119 (H2AK119ub) (Scheuermann et al., 2010). In addition, using the expressed H2A with Lys13 to Arg13 and Lys36 to Arg36 double mutations, the target product mono-ubiquitination of H2A at Lys15 (H2AK15ub) could also be obtained by *in vitro* enzymatic method (Mattiroli et al., 2012, 2014; Fradet-Turcotte et al., 2013; Li et al., 2014; Wang et al., 2016b; Wilson et al., 2016).

In recent years, chemical synthetic methods have been developed to obtain post-translationally modified histones with high uniformity in high efficiency (Maity et al., 2016). Basically, the chemical approaches for the manufacture of modified histones could be divided into three categories: cysteine-mediated protein modification, un-natural amino acid mutagenesis, and protein ligation. Each of these methods allows for the site-specific introduction of PTMs (or analogs of PTMs) into histones, which could then be biochemically assembled using the established methods into NCPs (Muller and Muir, 2015; Maity et al., 2017). It is worth noting that these methods have their own advantages and weaknesses, together making up a powerful toolbox for introducing PTMs into histones.

**Figure 2:**
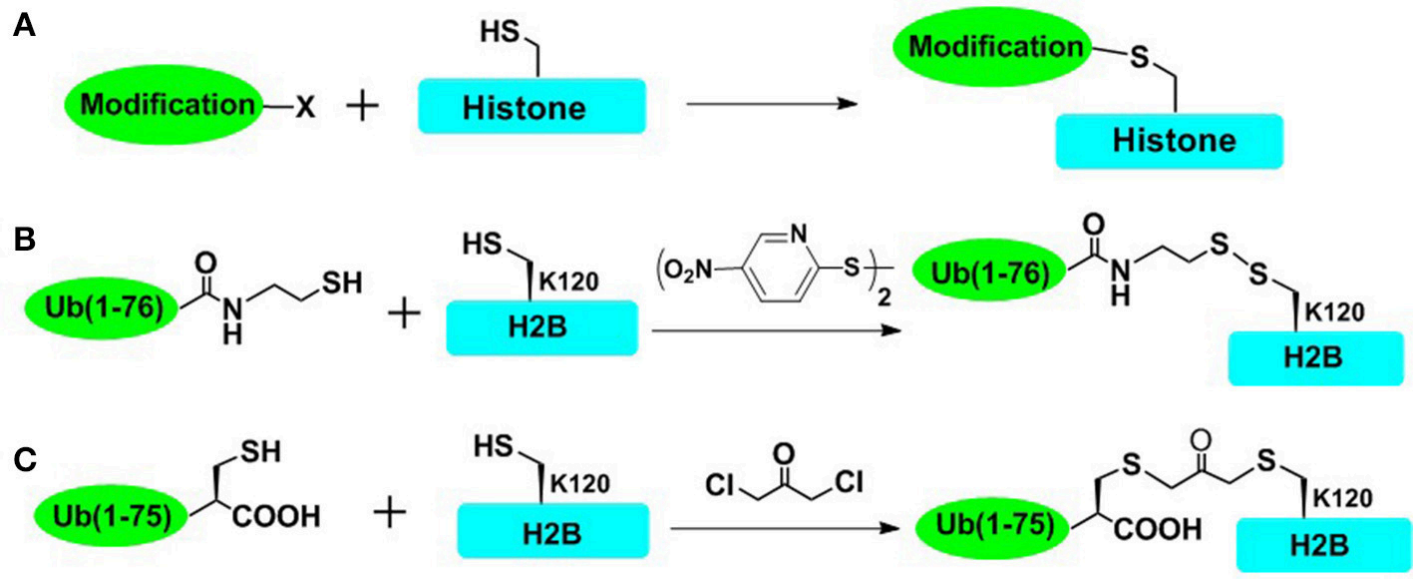
Cysteine-mediated protein modification approach. **(A)** The general concept of cysteine-mediated histone modification method. **(B)** Disulfide-directed ubiquitination of H2B. The sulfhydryl functional group was incorporated to the C terminal of recombinant ub and H2B-Lys120Cys mutant was expressed in Escherichia coli. The side-chain thiol group of Cys120 was activated by 2,2′-dithiobis(5-nitropyridine) (DTNP), and then cross-linked with the thiol of ub, affording the disulfide mimic of ubiquitinated H2B. **(C)** Dichloroacetone-directed ubiquitination of H2B. To prepare the site-specially ubiquitinated histone, H2B-Lys120Cys and Ub-Gly76Cys were expressed respectively and then crosslinked with dichloroacetone, affording the H2BK120ub mimic containing nonhydrolysable dichloroacetone.

To rapidly obtain post-translationally modified histones and reduce the workload of chemical synthesis, cysteine-mediated protein modification approach provides a straightforward route to introduce several PTM analogs into proteins (Figure 2; Chatterjee et al., 2010; Li et al., 2011; Long et al., 2014; Holt et al., 2015; Morgan et al., 2016; Wilson et al., 2016). The main principle of the cysteine-mediated protein modification method is to mutate the original Cys to Ala while mutating the amino acid residue (e.g., Lys and Arg) at the position of interest to a unique Cys, through standard recombinant expression methods. Subsequently, utilizing the high nucleophilicity of the mercapto group of Cys side chain, the site-selective addition reaction or alkylation reaction could take place between the mercapto group (within cysteine-containing histones) and specific small molecules or ubiquitin derivatives, ultimately affording the histone with site specific analogs of PTMs (Figure 2A). This method has been proved to be especially powerful for introducing analogs of Lys methylation, Lys acetylation, Arg methylation, and even Lys ubiquitination (Simon et al., 2007; Maity et al., 2016). Furthermore, cysteine-mediated protein modification has been used for elucidating structural aspects as well as crosstalk of NCPs. In one important example, Wolberger and coworkers studied the structural mechanisms of deubiquitination of H2BK120ub by deubiquitination enzyme SAGA (Morgan et al., 2016), where the mimic of H2AK120ub (mono-ubiquitination of H2B at Lys120) was prepared through the cysteine-mediated protein modification strategy. The complex of ubiquitinated NCP and SAGA was constructed, and the crystal structure of this complex was also deciphered. However, there are still many histone PTMs that could not be accessed by using this straightforward strategy. This approach could not mimic many PTMs such as Ser/Thr phosphorylation (Maity et al., 2016). Furthermore, it is also a challenge for this method to introduce multiple different PTMs into one histone protein.

Another powerful approach to prepare modified histones is direct genetic encoding of modified amino acids through the amber codon suppression technology (Davis and Chin, 2012). The key to this method is to evolve a dedicated tRNA/tRNA-synthetase pair for the modified amino acids of interest. The major advantage is that once the tRNAs/tRNA-synthetase evolution is successful, the synthesis of modified histones is straightforward and can be achieved in living cells (Lang and Chin, 2014). Many small PTMs (such as Lys methylation, Lys acetylation, and Lys phosphorylation) and functionalized unnatural amino acids (such as photolysine and azidonorleucine) could be directly introduced through genetic approach. However, introduction of multiple PTMs on the same histone protein or the introduction of large PTMs such as ubiquitination is still challenging for genetic strategies (Maity et al., 2016).

Protein ligation methods, including total chemical synthesis and semisynthesis approaches, have been widely used to prepare natural and modified histones in highly homogeneous fashion (Muller and Muir, 2015; Maity et al., 2016). Compared to the cysteine-mediated protein modification and un-natural amino acid mutagenesis, protein ligation methods provide more flexibility in terms of PTM types and PTM number that could be introduced into one histone protein. Both total chemical synthesis and semisynthesis approaches apply native chemical ligation (NCL) or modified versions of NCL to assemble target histones or modified histones from multiple synthetic and/or recombinant peptide segments (Dawson et al., 1994; He et al., 2003; Kent, 2009; Huang and Liu, 2015; Bondalapati et al., 2016; Huang et al., 2016; Lee and Li, 2016; Medini et al., 2016). Generally, both NCL and modified versions of NCL were conducted between the thioester segment and the Cys segment (Zheng et al., 2011; Holt and Muir, 2015; Raibaut et al., 2016; Bondalapati et al., 2017; Li and Dong, 2017; Tan et al., 2017).

**Figure 3:**
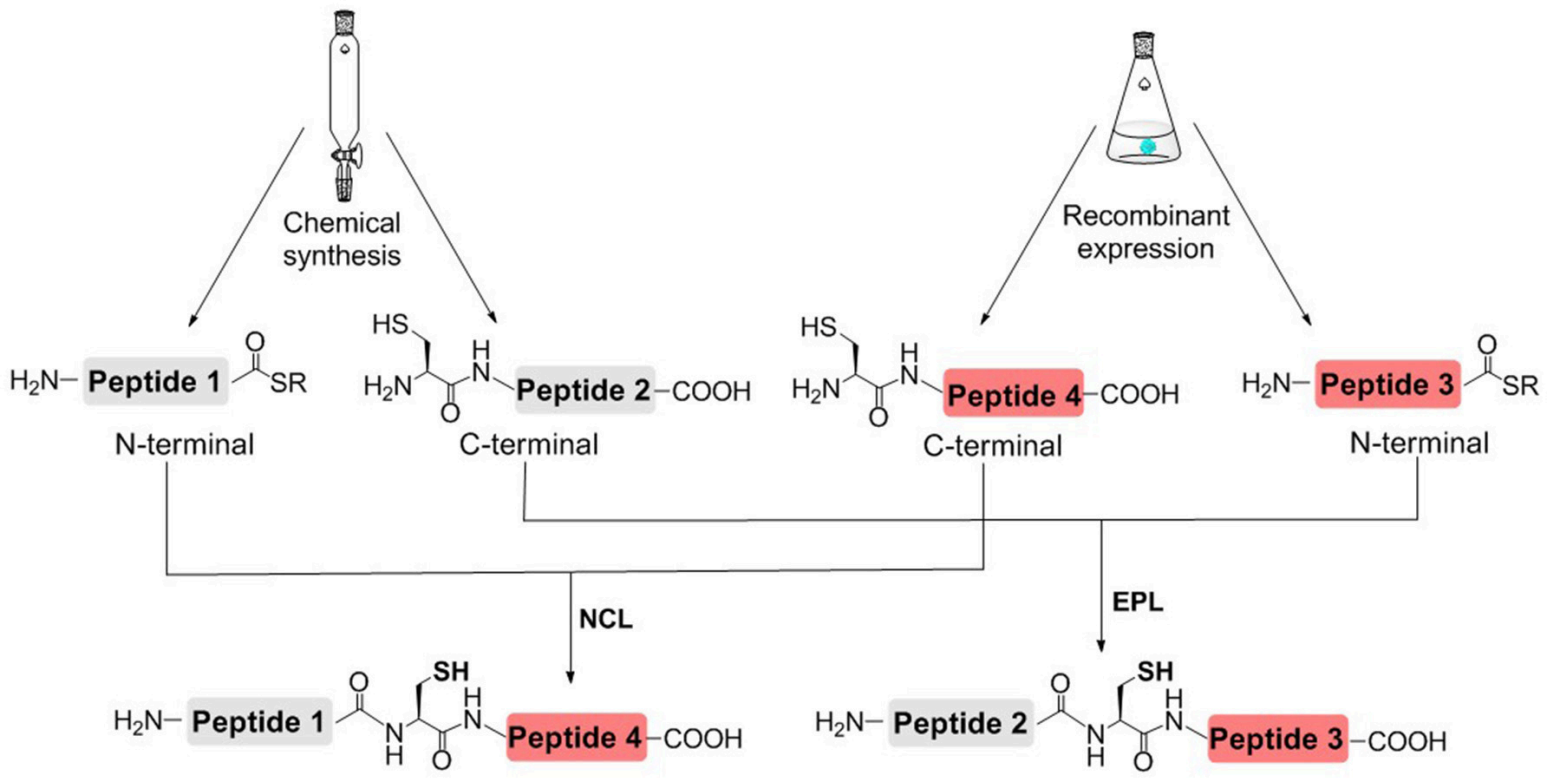
Histone semisynthesis by native chemical ligation (NCL) and expressed protein ligation (EPL). Histone fragments containing C-terminal α-thioester or N-terminal Cys residues are prepared by solid phase peptide synthesis (SPPS) or recombinant expression.

In order to reduce the workload of chemical synthesis, protein semisynthesis has been used for the synthesis of site-specifically modified histones. For protein semisynthesis, the thioester segment or the Cys segment without PTM, was obtained by recombinant expression. The other segment, which has one or multiple PTMs, was prepared by solid phase peptide synthesis (SPPS) (Figure 3; Muir et al., 1998; Holt and Muir, 2015). The biologically expressed part of target protein could be obtained not limited by the length or type of peptide sequence and thus efficiently afford the long sequence of peptide segment. On the other hand, SPPS could enable flexible modification of chemically synthesized segments, that is, site-specific installation of one or multiple PTMs at any desired site of protein.

Protein semisynthesis is suitable for the preparation of modified histones with C-terminal or N-terminal modifications (McGinty et al., 2008, 2009; Fierz et al., 2012; Bi et al., 2016; Maity et al., 2016). However, it may be challenging for the semisynthesis methods to prepare modified histones with modifications in the middle of the protein sequence or multiple modifications at both C-terminal and N-terminal.

## Total chemical synthesis applied to histones with PTMs

The total chemical synthesis takes advantage of solid-phase peptide synthesis (SPPS) to obtain histone segments with site-specific modifications and NCL to ligate one peptide with C-terminal peptide thioester and peptide with an N-terminal cysteine residue. Subsequently, two or more segments are assembled to achieve full length modified protein through convergent or sequential strategies. The total chemical synthesis approach theoretically permits installation of multiple different post-translational modifications at any desired site at histone (Wang et al., 2013; Muller and Muir, 2015). In recent years, combined with the improvement of synthetic method to generate histone peptide fragments, the introduction of peptide hydrazide strategy and desulfurization strategy (Wan and Danishefsky, 2007; Fang et al., 2011, 2012; Zheng et al., 2013a,b; Muller and Muir, 2015; Qi et al., 2015; Tang et al., 2016; Jin et al., 2017; Li et al., 2017b; Li Y. T. et al., 2017; Maity et al., 2017), it has substantially reduced the time and effort to obtain homologous histones for biochemical and structure study. This section would explore the more recent advances for the total chemical synthesis of histone with specific PTMs.

## The total chemical synthesis of H2A with PTMs

In 2016, Okamoto and coworkers conducted the first chemical synthesis of a variety of post-translationally modified histones H2A through Fmoc based SPPS and sequential NCL utilizing *N-*acylurea chemistry (Hayashi et al., 2016). H2A was divided into three peptide segment and the N-terminal cysteine of segment H2A (47–85) was temporarily protected with thiazolidine (Thz) to avoid self-ligation. *N-*Acylbenzimidazoline (Nbz) was used as a C-terminal thioester precursor. Two steps of NCL was performed between H2A peptide segments in N-to-C form, followed by radical desulfurization to obtain natural H2A and H2A with three PTMs (S1ph, K75me2, and K119ac). The synthetic H2As was assembled into nucleosomes, and the thermal stability test revealed that the three post-translational modifications have no effect on nucleosomal stability.

The first chemical synthesis of phosphorylated histone H2A at Tyrosine 57 residue (H2AY57ph) was achieved by Brik and coworkers (Jbara et al., 2016). Phosphorylated tyrosine amino acid was coupled into H2A peptide through Fmoc SPPS. The using of protecting group and thioester precursor of H2A peptide segments were the same as Okamoto group. Three H2A peptide segments were assembled via sequential NCL followed desulfurization to furnish H2AY57ph in 25% yield. Synthetic H2AY57ph and H2BK120ub were assembled into nucleosomes and subjected to SAGA deubiquitination assay. The biochemical results showed that phosphorylation of H2A57 reduced the enzyme activity of SAGA toward H2AK120ub. The structure of SAGA-H2BK120ub complex showed that 57 tyrosine residue of H2A lies at the interface between the SAGA DUB module and its contact surface with histones H2A and H2B (Morgan et al., 2016). These results explained that the phosphorylation of this site affects SAGA DUB activity. The Okamoto and coworkers also completed the total chemical synthesis of H2AY57ph recently and revealed that H2AY57 tyrosine phosphorylation changes the interaction between H2A-H2B dimers. The natural nucleosomes structure showed that H2AY57 and H2B αC-helix in very close sites, Y57 phosphorylation may thus affect the stability of H2A-H2B dimer (Sueoka et al., 2017).

**Figure 4:**
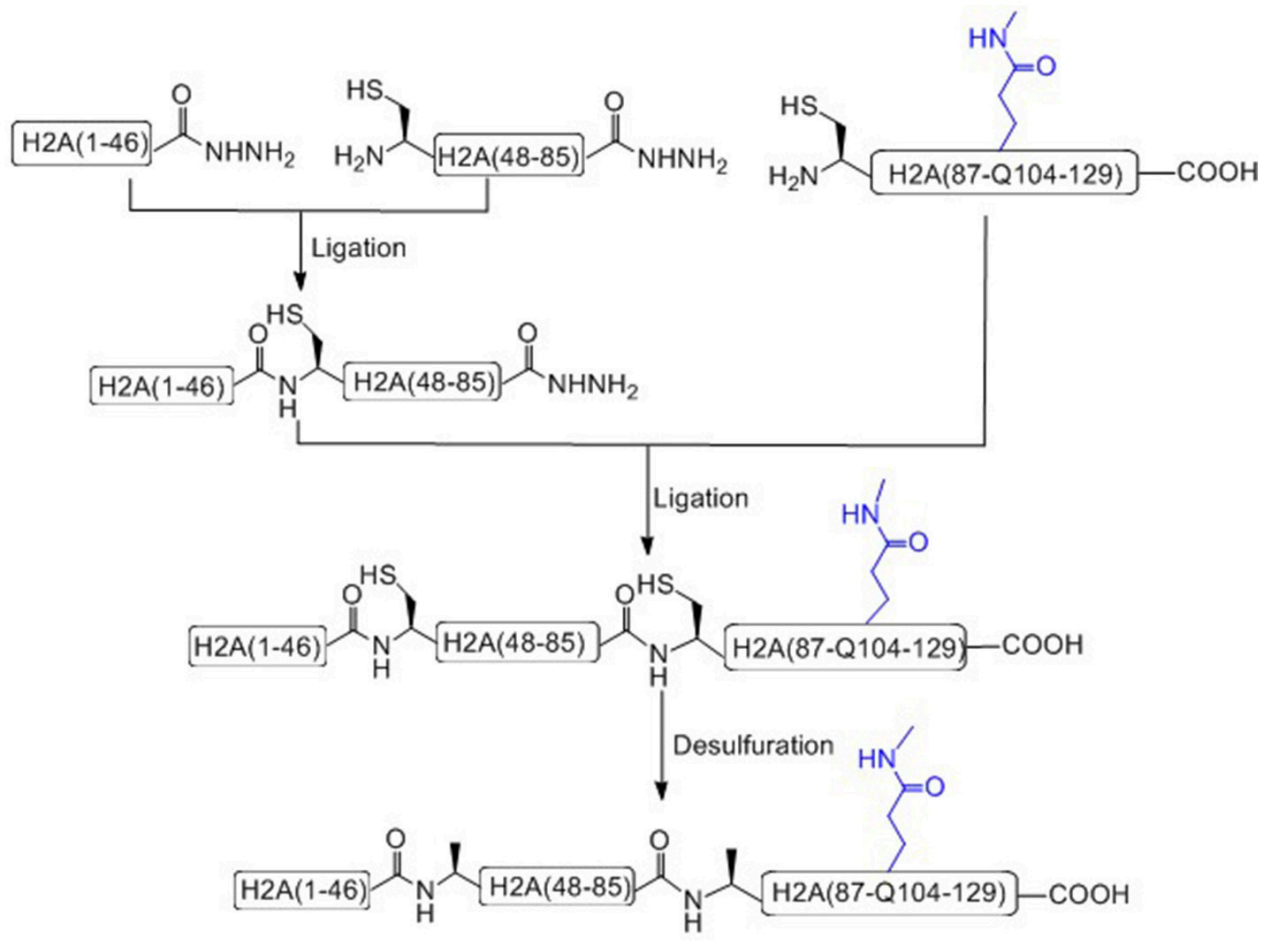
Total chemical synthesis of H2AQ104me1 from three H2A segments.

Liu and coworkers recently has achieved the total chemical synthesis of H2AQ104me1 (Figure 4; He et al., 2017). Unlike others, in the prepared peptide fragments containing C-terminal hydrazide, the N-terminal Cysteine residue can be used without any protecting group (Tang et al., 2015; Pan et al., 2016; Wang et al., 2016a). It is worth to notice that, during the key methylated polypeptide synthesis, commercial available Fmoc-Glu(Allyl)-OH was used as the precursor building block of Fmoc-Gln(me1). After coupling the last amino acid, the Allyl protecting group of glutamate was removed on resin and condensed with methylamine to afford mono-methylated glutamic acid at the 104 residue site (Huang et al., 2013).

In addition, Li and coworkers have recently developed a new, highly efficient desulfurization strategy for the successful synthesis of native H2A (Jin et al., 2017). This method was performed based on TCEP-NaBH_4_ instead of the normal radical for desulfurization.

## The total chemical synthesis of H2B with PTMs

**Figure 5:**
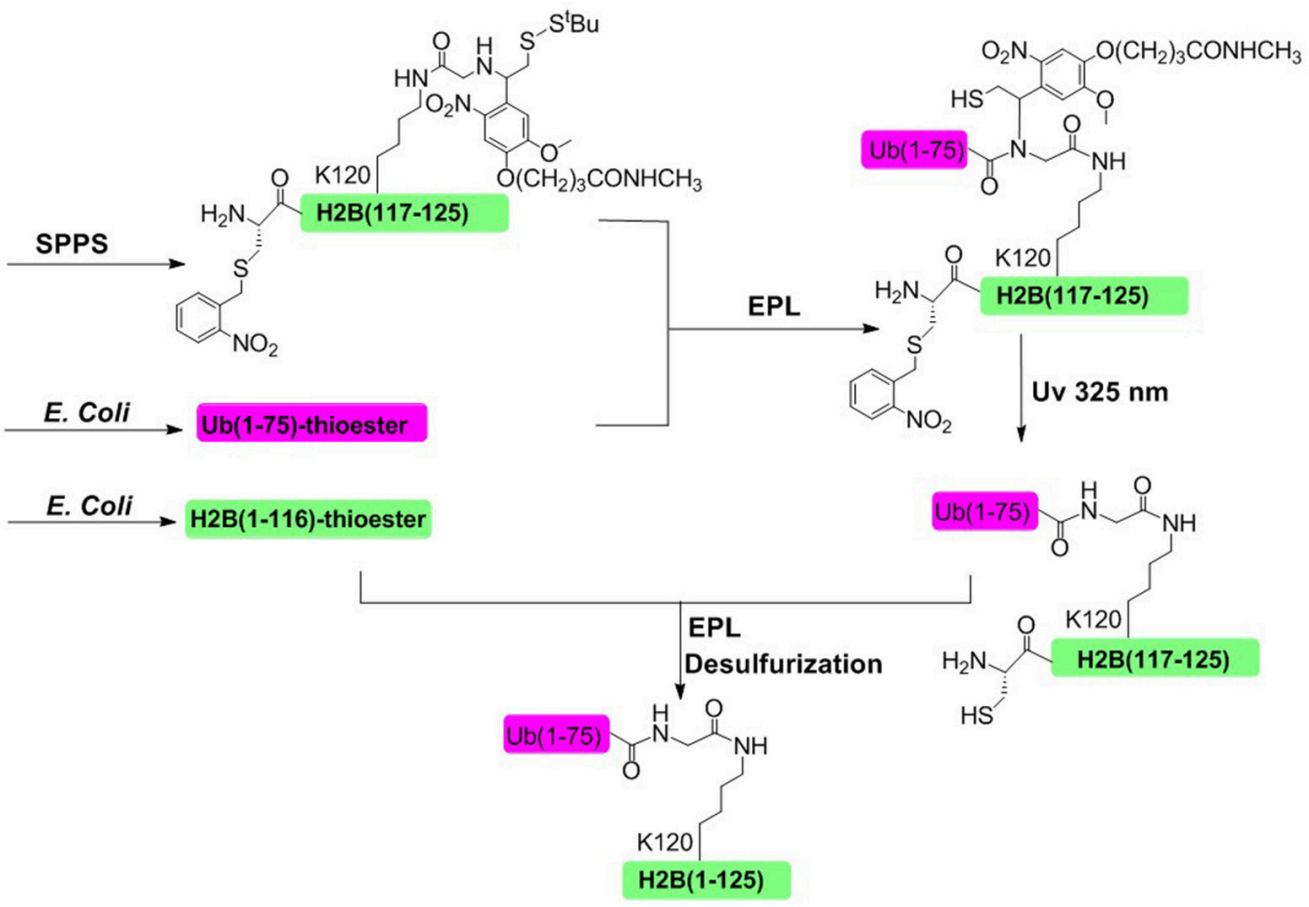
Photolabile auxiliary-mediated semisynthesis of H2BK120ub. The H2BK120ub was divided into three peptide segments. ub (1–75)-thioester and H2B (1–116)-thioester were obtained through recombinant method. H2B (117–125) segment containing the photolytic auxiliary was prepared through Fmoc SPPS.

In 2008, Muir and coworkers achieved the semisynthesis of H2BK120ub through the combination of EPL and a thiol-containing, photolytically removable, ligation auxiliary (Figure 5; Chatterjee et al., 2007; McGinty et al., 2008). The first ligation was conducted between an ubiquitin a-thioester and a glycine derivative attached to the side-chain of Lys120. Then the photolabile auxiliary was cleaved by photolysis, restoring the native ubiquitin moiety attached to Lys 120 residue. The second ligation was conducted between the H2B (1–116)-thioester and ubiquitinated H2B (117–125). Upon completion of the second ligation and desulfurization, the native H2BK120ub was obtained with an overall isolated yield of 20%.

In 2013, Brik and coworkers achieved the chemical synthesis of H2BK34ub for the first time by ligation of four H2B peptide segments and ubiquitin thioester (Siman et al., 2013). Different from methylation, acetylation, phosphorylation and other modifications, the ubiquitination modification is formed between the ubiquitin C-terminal and the histone-specific lysine side chain residues through an isopeptide bond. To construct the isopeptide bond between H2B and ubiquitin, γ-thiolated lysine was introduced into the K34 position because the γ-thiolated lysine own similar ligation reactivity as cysteine. The thiol group could be removed by desulfurization after H2Bub skeleton construction. It should emphasis that the amino group of Lys57 side chain in segment H2B (21–57) was protected with a photo-sensitive 6-nitroveratryloxycarbonyl (Nvoc) group to prevent lactamization in the ligation step, while the cysteine of segment H2B (59–96) N-terminal was protected with thiazolidine (Thz). Peptide precursor thioesters prepared by Nbz and hydrazide methods were ligated by the convergent synthesis strategies, followed by free radical based desulfurization and *in situ* removal of Nvoc to give the desired H2BK34ub.

Brik and coworkers also compared the efficiency of the different synthetic approaches for a variety of modified H2B (H2BK34ub, H2BS112GlcNAc, H2BK120ub, and H2B-S112GlcNAc-K120ub), and concluded that convergent strategy for the synthesis of most of these complex targets was more efficient than one-pot approach (Seenaiah et al., 2015). Another work reported by this group presented the total chemical synthesis of H2B on the resin for the first time using the Rink-amide linker strategy through solid-phase chemical synthesis (SPCL) (Jbara et al., 2014). This strategy performs a higher number of chemical steps and with a greater diversity of chemical transformations. Ligation and desulfurization steps could be processed on solid phase for avoiding separation and lyophilization procedures, and then native H2B were cut off from resin with TFA cleavage agent.

**Figure 6:**
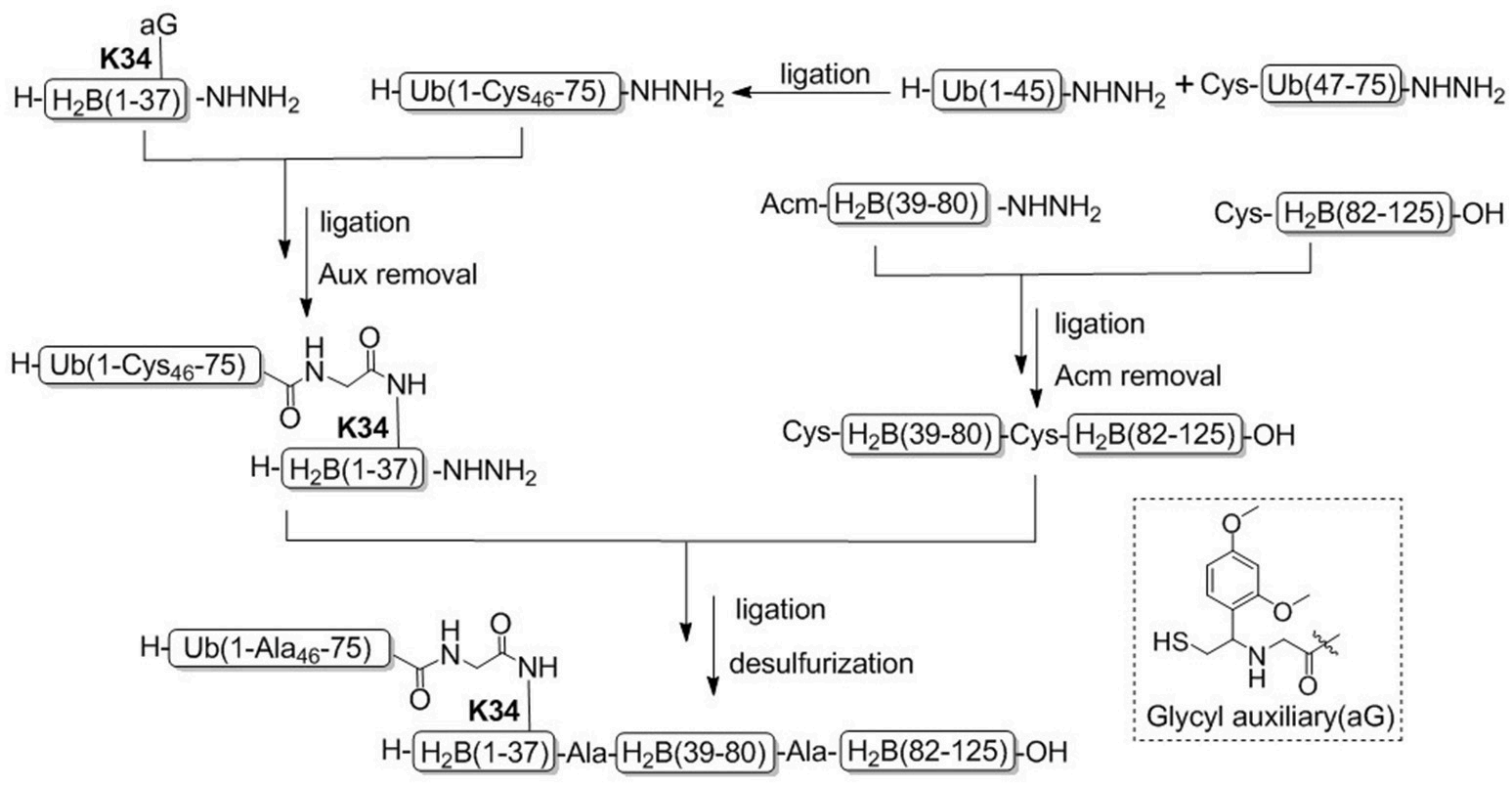
Total chemical synthesis of H2BK34ub by using acid-cleavable auxiliary-mediated ligation of peptide hydrazides.

Recently, Tian and coworkers achieved another example for the synthesis of H2BK34ub by using acid-cleavable auxiliary-mediated ligation of peptide hydrazides (Figure 6; Li et al., 2017a). Acid-sensitive auxiliary is condensed into the side chain amino group of Lys34 during solid phase synthesis, which is capable of producing a NCL-like reaction with ubiquitin hydrazide. Then site specific ubiquitination on H2B lysine 34 was achieved by removing the auxiliary group with TFA cleavage reagent. Synthetic H2BK34ub was assembled into nucleosomes for single-particle cryo-electron microscopy (cryo-EM) imaging. The cryo-EM structure exhibited that two flexible ubiquitin domains protrude between the DNA chains of the nucleosomes, and the DNA strands around the H2BK34 sites shift and provide more space for ubiquitin prominence. This study developed an alternative strategy for the preparation of ubiquitinated modified nucleosomes, which provided an effective tool for the further study of the structural mechanism of ubiquitination of nucleosomes at other different histone sites. The acid-cleavable auxiliary-mediated native isopeptide bond construction strategy has also been successfully applied to the chemical synthesis of H2BK120ub (Qi et al., 2017b).

Besides, Liu and coworkers have developed a novel crypto-thioesters based on peptide *o*-aminoanilides for protein chemical synthesis, and native H2B histone was total chemical synthesized with this method (Wang et al., 2015).

## The total chemical synthesis of H3 with PTMs

In 2010, the Liu group achieved the first semisynthesis of native H3 protein and H3K4me2 through a thioacid capture ligation strategy (TCL) (Zhang et al., 2011). The peptide ligation reactions based on the TCL were accomplished in about 30 min, affording the target H3 and H3K4me2 with ligation yields of about 70% based on RP-HPLC analysis. The first total chemical synthesis of acetylated histone H3 on Lys56 (H3K56ac) was prepared by Ottesen and coworkers in 2011 (Shimko et al., 2011). Three H3 peptide segments were ligated in C-to-N order, followed by desulfurization to furnish H3K56ac in 7% yield. The synthetic peptides were assembled into nucleosomes and shown to increase the binding affinity of the DNA factor LexA by increasing DNA unwrapping. The similar approach was also successfully applied to the total chemical synthesis of H3Y41ph and H3Y41ph-K56ac histone by Poirier (Brehove et al., 2015). The phosphorylated and acetylated histone H3 was used for DNA accessibility assay, and the result shows that histone phosphorylation near the DNA entry-exit region of the nucleosome increases DNA unwrapping and accessibility. When phosphorylation and histone acetylation occur simultaneously, the effect is further enhanced.

Aimoto and coworkers completed the first total chemical synthesis of histone H3 using Cys-Pro ester (CPE) self-activated units as thioester precursors (Toru et al., 2013). It is interesting to notice that the “ligation” between H3 (1–43) and H3 (44–135) was not accomplished by native chemical ligation, but through the condensation of the glycine amino group with the thioester. Non-coupled nucleophilic groups, such as amino groups or thiol group, were protected with an acid-sensitive Boc group or dithiothreitol (DTT)-removable methylthio group. Combining the thioester methods and NCL methods offers more flexibility in choosing condensation sites.

**Figure 7:**
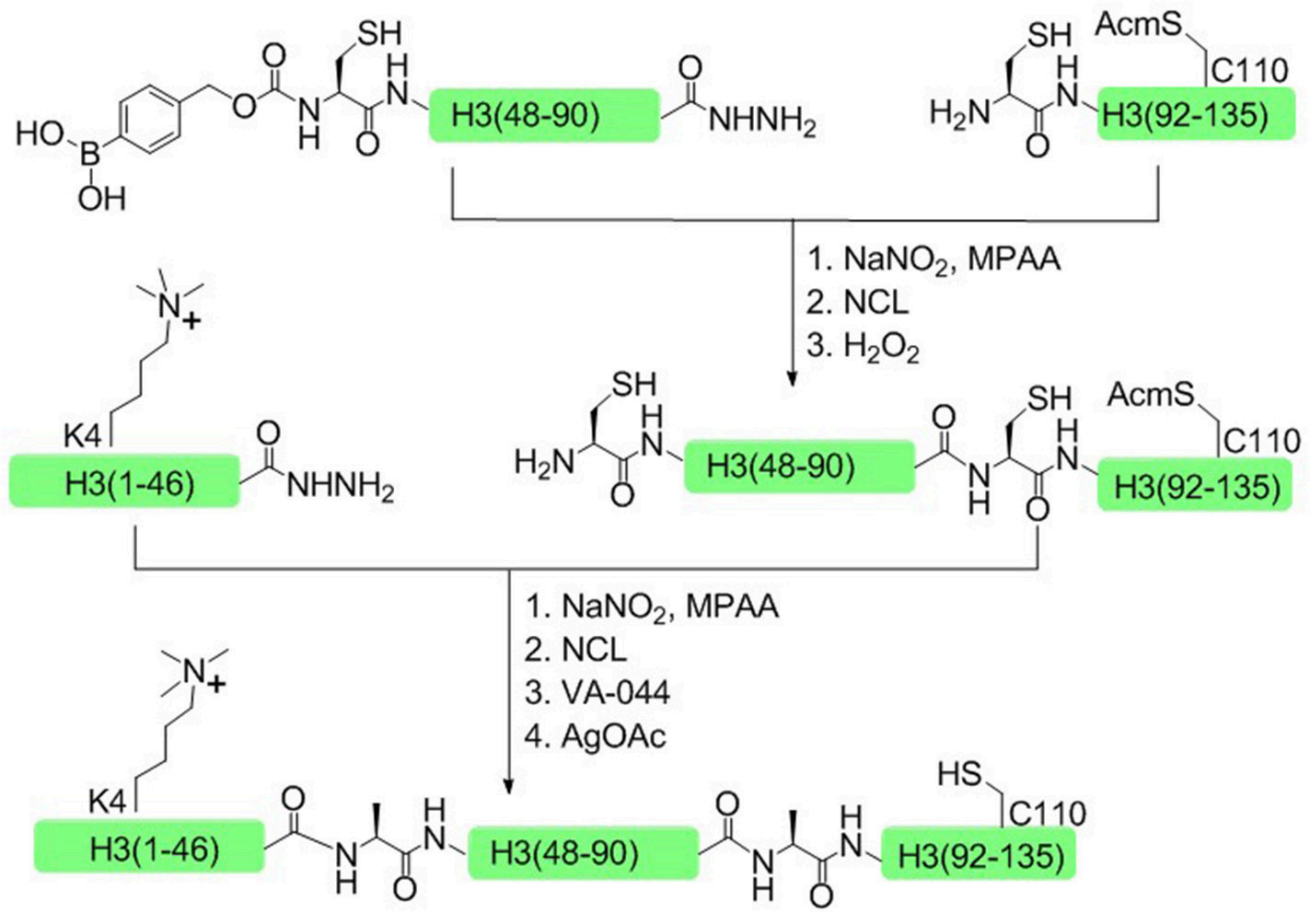
Total chemical synthesis of H3K4me3 by using H_2_O_2_-controlled one-pot native chemical ligation of peptide hydrazides.

In 2014, Liu and coworkers developed another one-pot ligation strategy based on peptide hydrazide, which required only a single step of purification to achieve the desired product (Li et al., 2014). This approach was successfully used to synthesize H3K4me3 and H4K16ac histone. Taken H3K4me3 as an example, histone H3 was divided into three polypeptide segments and the peptide hydrazides were synthesized by Fmoc SPPS. The N terminal cysteine of the middle fragment H3 (46–91) was temporarily sheltered with *p-*boronobenzyloxycarbonyl (Dobz) group that can be removed easily by H_2_O_2_ to process the next chemical ligation step, thus circumventing the necessary for the isolation of intermediate products (Figure 7).

Very recently, Li and coworkers reported their endeavors toward the first total chemical synthesis of bivalently modified H3 bearing Lys56 acetylation and Lys122 ubiquitination (H3K56ac-K122ub) (Qi et al., 2017a). The two site-specific PTMs of histone H3 were speculated to synergistically facilitate the formation and deposition of (H3-H4)_2_ tetramers. The acetylated lysine was directly condensed into H3 (47–90) segment at Lys56 site to furnish acetylated H3 peptide fragment, while isopeptide bond of K122ub was constructed using acid-cleavable auxiliary in H2BK34ub synthesis. Three H3 peptide segments were assembled and subsequently desulfurized to obtain bivalently modified H3.

Brik and coworkers recently synthesized H3K79me3 and its analogs by convergent chemical synthesis approach. They further identified KDM4D lysine demethylase as a potential regulator of H3K79me3 (Jbara et al., 2017).

## The total chemical synthesis of H4 with PTMs

It was mentioned above that H3K4me3 and H4K16ac histone were synthesized by one-pot hydrazide based ligation method in 2014 (Li et al., 2014). As far as we know, this is the first successful synthesis of histone H4.

In 2016, Ottesen and coworkers developed a HMBA linker resin to achieve the chemical ligation and desulfurization in the solid-liquid mixed phase (Yu et al., 2016). In this way, many intermediate separation steps were circumvented, which contributes to save time and improve the efficiency of protein synthesis. Finally, the target protein was obtained by removing the HMBA linker with trifluoroacetic acid (TFA) cleavage. The strategy was successfully applied to the synthesis of triple-acetylated H4 (K5ac, K12ac, and K91ac). Similar work including the TFA-labile Rink linker developed by the Brik group was mentioned above for the synthesis of H2B (Jbara et al., 2014).

Recently, Hojo and coworkers reported a one-pot polypeptide ligation strategy for the chemical synthesis of native H4 synthesis based on pH-sensitive protective groups (Asahina et al., 2017). This strategy takes advantages of two orthogonal thioester precursors–Cysteinylprolylester (CPE) and *N-*Alkycysteine (NAC), which could be converted to the corresponding thioesters by adjusting to different pH during the ligation process. Moreover, this work has also developed a protective group of Asp side chain carboxyl groups for the native chemical ligation of Asp with Cys, which prevents intramolecular cyclization reaction in the Asp-involved ligation step.

Because almost all H4 histones were synthesized based on the three-segment ligation method, Brik and coworkers recently reported a double-fragment synthesis of H4. H4 was divided into two segments H4 (1–37) and H4 (38–102), in which the 69 site Ala of H4 (38–102) were temporarily mutated into Cys residue for introducing an easily removable solubilizing tag to assist the synthesis and ligation of hydrophobic peptides. After two segments ligation, palladium chemistry was used to remove Cys-protecting groups in a one pot manner, followed by radical desulfuration to afford native H4. This strategy combined with two-segment ligation and one-pot palladium-mediated deprotection was expected to be applied to the synthesis of H4 or derived histone with higher efficiency (Maity et al., 2017).

## Summary and outlook

**Figure 8:**
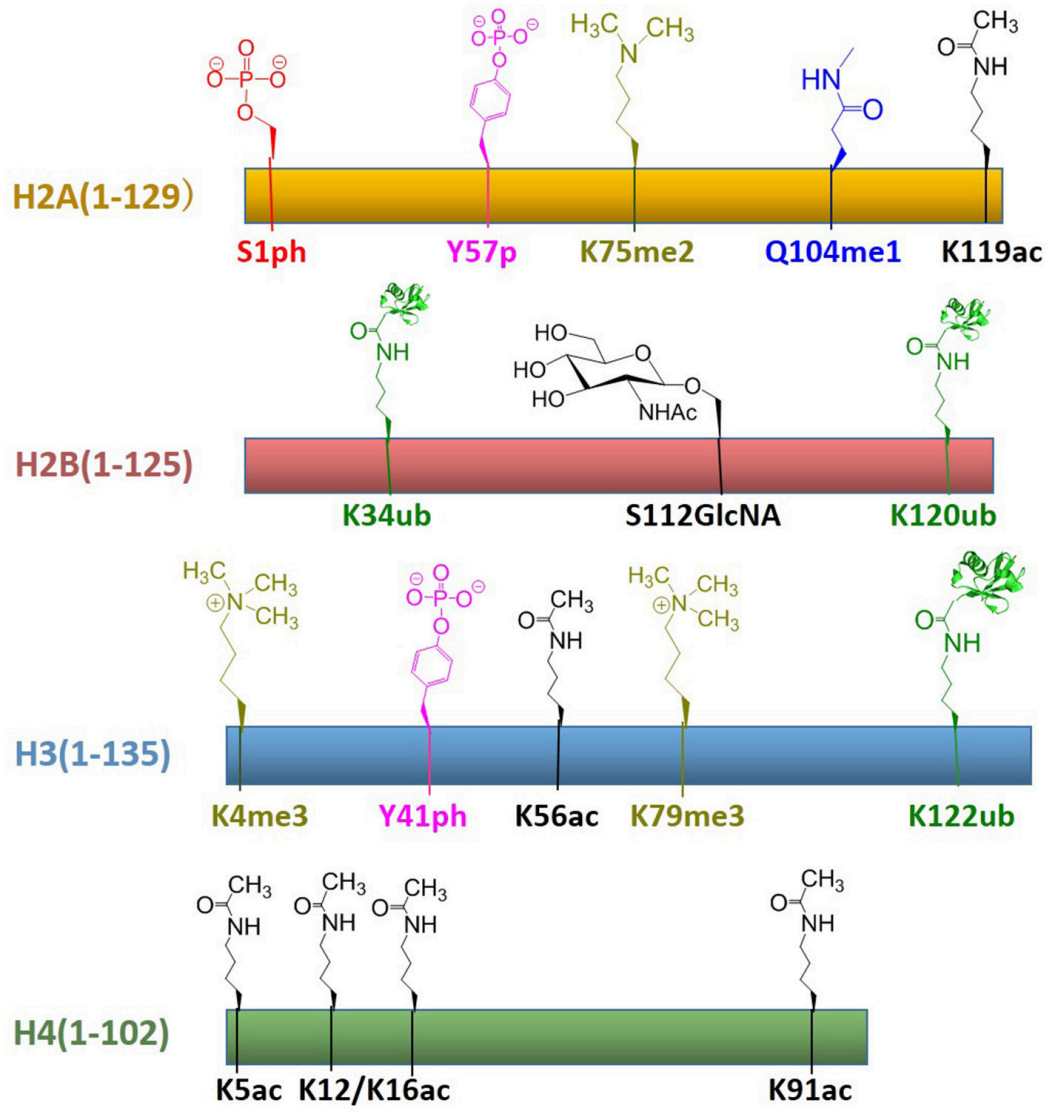
Summary of established the total chemical synthesis strategies to assemble the four canonical histones with post-translational modifications. Rectangles indicate the histone primary sequences, H2A (yellow), H2B (red), H3 (blue), and H4 (green). Post-translational modifications (PTMs) that have been installed by chemical ligation techniques are annotated above each histone in different color: serine phosphorylation (red), tyrosine phosphorylation (pink), lysine di-/tri-methylation (yellowish green), glutamine mono-methylation (blue), lysine ubiquitination (green), serine GlcNAcylation (brown), lysine acetylation (black).

In recent years, protein total chemical synthesis and semi-synthesis have been used to generate an array of post-translationally modified histones, including methylation, acetylation, GlcNAcylation, phosphorylation, ubiquitination, etc. These reagents enabled the study of “histone code”—process for the installation, recognition and removal of these marks. These efforts significantly improved our understanding of post-translationally modified proteins and provided synthetic access to histones with modifications at most regions of its primary sequence. All histones with post-translational modifications obtained by total chemical synthesis are summarized in Figure 8. There are still many key areas for improvement in chemical synthesis of histones, such as the development of efficient strategies for the synthesis of histone peptides derived from both domain and insoluble domains, suitable protection/deprotection methods as well as the rapid assembly of multiple PTMs for histone analogs (Nguyen et al., 2014). As the synthetic methods become more sophisticated, we believe that the manufacture of modified histones will be even more convenient. Then the biggest challenge at this point might be how to use these reagents to decipher chromatin complexity and how to incorporate these building blocks into a larger array of nucleosomes closer to native chromatin. Therefore, in the chromatin area, chemists still have many opportunities to develop novel methodologies for understanding epigenetic languages and even beyond the world of histones.

## Author contributions

All authors listed have made a substantial, direct and intellectual contribution to the work, and approved it for publication.

### Conflict of interest statement

The authors declare that the research was conducted in the absence of any commercial or financial relationships that could be construed as a potential conflict of interest.
